# Supplementary material for: Non-Alcoholic Steatohepatitis Decreases Microsomal Liver Function in the Absence of Fibrosis
Source: Biomedicines. 2020 Nov 27;8(12):546. doi: 10.3390/biomedicines8120546 (PMC7760673; doi:10.3390/biomedicines8120546)
Supplement: Supplementary file 1 [file biomedicines-08-00546-s001.pdf]

Supplementary materials

Table 1. S: P values for patient characteristics for NAFLD-F.

**Value**	**P (NAFLD-F vs. noNAFLD)**	**P (NAFLD-F vs. NAFL)**	**P (NAFLD-F vs. NASH-noF)**
Age (yrs)	<0.0001*	0.022*	0.007*
Gender (female)	<0.00001*	0.012*	0.021*
Smoking (non-smoker)	0.886	0.525	0.131
BMI (kg/m2)	0.170	0.604	0.690
Waist (cm)	<0.00001*	0.002*	<0.0001*
AST (U/L)	<0.00001*	<0.00001*	<0.00001*
ALT (U/L)	<0.00001*	<0.0001*	0.012*
GGT (U/L)	<0.00001*	<0.00001*	<0.00001*
PLT (10E9/L)	<0.0001*	0.002*	<0.00001*
Tot chol (mg/dL)	0.027*	0.011*	0.001*
HDL (mg/dL)	<0.00001*	0.013*	0.001*
TG (mg/dL)	0.007*	0.502	0.903
LDL (mg/dL)	0.156	0.052	0.016*
Tot bili (mg/dL)	<0.001*	0.018*	0.007*
HbA1c (%)	<0.00001*	<0.0001*	<0.00001*
Alb (g/dL)	0.049*	0.015*	0.001*
INR	<0.0001*	0.001*	<0.0001*
Steatosis	<0.00001*	<0.00001*	<0.0001*
Inflammation	<0.00001*	<0.00001*	<0.00001*
Ballooning	<0.00001*	<0.00001*	<0.00001*
NAS	<0.00001*	<0.00001*	<0.0001*
USS	<0.00001*	0.018*	0.277
ABTpeak	<0.00001*	<0.00001*	<0.0001*
ABTcum	<0.00001*	<0.00001*	<0.001*

P values for the characteristics of patients with non-alcoholic fatty liver disease and significant fibrosis (NAFLD-F) versus patients without NAFLD (noNAFLD), patients with non-alcoholic fatty liver (NAFL) and patients with non-alcoholic steatohepatitis without significant fibrosis (NASH-noF). P-value is calculated between different groups with * indicating statistical significance (<0.05). BMI, body mass index; AST, aspartate aminotransferase; ALT, alanine aminotransferase; GGT, gamma glutamyl transpeptidase; PLT, platelets; tot chol, total cholesterol; HDL, high density lipoprotein cholesterols; TG, triglycerides; LDL, low density lipoprotein cholesterols; tot bili, total bilirubin; HbA1c, haemoglobin A1c; Alb, albumin; INR, international normalized ratio; NAS, NAFLD activity score; USS, ultrasound steatosis score; ABTpeak, aminopyrine breath test peak value; ABTcum, aminopyrine breath test cumulative value.
